# Comparative Quality Control of Titanium Alloy Ti–6Al–4V, 17–4 PH Stainless Steel, and Aluminum Alloy 4047 Either Manufactured or Repaired by Laser Engineered Net Shaping (LENS)

**DOI:** 10.3390/ma13184171

**Published:** 2020-09-19

**Authors:** Noam Eliaz, Nitzan Foucks, Dolev Geva, Shai Oren, Noy Shriki, Danielle Vaknin, Dimitry Fishman, Ofer Levi

**Affiliations:** 1 Biomaterials and Corrosion Lab, Department of Materials Science and Engineering, Tel-Aviv University, Ramat Aviv, Tel Aviv 6997801, Israel; nitzanfoucks2@gmail.com; 2 Materials Science and Engineering Division, Depot 22, Israel Air Force, P.O. Box 02538, Tel Aviv, Israel; orenshai20@gmail.com (S.O.); noya.shr@gmail.com (N.S.); daniellva199658@gmail.com (D.V.); dimitry.fishman@gmail.com (D.F.); ofemi6674@gmail.com (O.L.); 3 Israel Ministry of Defense, Hakirya, Tel Aviv 61909, Israel; dolev24@yahoo.com

**Keywords:** additive manufacturing (AM), directed energy deposition (DED), laser engineered net shaping (LENS), titanium, aluminum, stainless steel, micro-computed tomography (μ-CT), surface roughness, microstructure, porosity

## Abstract

Additive manufacturing attracts much interest for manufacturing and repair of structural parts for the aerospace industry. This paper presents comparative characterization of aircraft items made of Al 4047 alloy, Ti-6Al-4V alloy, and 17-4 precipitation hardened (PH) (AISI 630) stainless steel, either manufactured or repaired by laser engineered net shaping (LENS). Chemical analysis, density, and surface roughness measurements, X-ray micro-computed tomography (μ-CT) analysis, metallography, and micro-hardness testing were conducted. In all three materials, microstructures typical of rapid solidification were observed, along with high density, chemical composition, and hardness comparable to those of the counterpart wrought alloys (even in hard condition). High standard deviation in hardness values, anisotropic geometrical distortion, and overbuild at top edges were observed. The detected defects included partially melted and unmelted powder particles, porosity, and interlayer lack of fusion, in particular at the interface between the substrate plate and the build. There was a fairly good match between the density values measured by μ-CT and those measured by the Archimedes method; there was also good correlation between the type of defects detected by both techniques. Surface roughness, density of partially melted powder particles, and the content of bulk defects were significantly higher in Al 4047 than in 17-4 PH stainless steel and Ti-6Al-4V alloy. Optical gaging can be used reliably for surface roughness measurements. The implications of these findings are discussed.

## 1. Introduction

The industry of additive manufacturing (AM) of metals has been growing rapidly in three directions—new AM machines with enhanced capabilities, compatible raw materials, and new applications [1]. Since AM enables the formation of parts with highly complex geometries, reduced mass, and sometimes with a “tailored” chemical composition; many industries, including the aerospace industry, now seek to incorporate AM into their production lines. The aircraft industry, for example, is interested both in manufacturing of parts from scratch and in repair of existing parts during maintenance. AM may allow for production of aircraft parts that are currently costly to produce. Some manufactures have already demonstrated the great capability of AM to reduce both the time and cost of production of such parts [2,3]. Regarding maintenance, aircrafts’ mechanical parts such as those placed in the engine or the landing gear, erode over time, losing material and failing to meet their critical dimensional requirements. Some AM processes, such as directed energy deposition (DED) [4,5,6,7], enable the addition of material to existing parts without harming them. This can greatly reduce maintenance costs without risking flight safety. One of the major DED technologies is laser engineered net shaping (LENS) [8], developed by Sandia National Laboratories and licensed in 1997 to Optomec, Inc. (Albuquerque, NM, USA). 

The thermal history of parts fabricated by the LENS process is determined by three parameters. (1) High cooling rates. LENS employs a high power laser (typically, 500 W–3 kW). This, combined with a small melt pool and a high scanning speed, give rise to high cooling rates (ca. 1000–5000 °C/s) [4]. Consequently, a fine grain structure is typically formed [9], resulting in excellent static and dynamic mechanical properties. (2) Absence of pre-heating in typical LENS systems. Most LENS systems do not have the capability of preheating the printing substrate plate [10]. Consequently, the microstructure of the deposited material might change along the vertical (build) direction, resulting in anisotropic mechanical properties. This may be overcome either by in-house installation of a pre-heating system or using accessories such as melt pool sensor (MPS) [11,12]. (3) Remelting and reheating. LENS, like other AM processes, fabricates the part by adding consecutive powder layers on top of each other and melting them together. With each addition of a new layer, the layer directly beneath the melt pool is remelted, and some layers beneath it are reheated. This can cause secondary changes in the microstructure of the part, allowing for the rise of anisotropic properties [4].

Various common defects in materials deposited by AM processes might adversely affect the properties of the final part. These include: (1) Depletion of volatile alloying elements (e.g., Mg and Zn in aluminum alloys) [13,14]. (2) Porosity and lack of fusion [1,14,15,16,17,18,19], which negatively affect the mechanical properties, in particular the fatigue resistance [20]. (3) Hot cracking. (4) High surface roughness [14]. Surface roughness has a critical effect on the mechanical properties of the printed part, especially fatigue. Surface roughness of ca. 200 µm can lower the fatigue strength by 20–25%, depending on the AM process [20].

In this work, three materials were deposited by LENS: 17-4 (AISI 630) precipitation hardened (PH) stainless steel, Ti-6Al-4V alloy, and aluminum 4047 alloy. Ti-6Al-4V is widely used in the aerospace industry due to properties such as low density, high specific strength, excellent specific stiffness, high service temperature, and excellent corrosion resistance. This alloy has a dual microstructure, which consists of a fine-grained hexagonal close-packed (hcp) α-phase and a distributed body-centered cubic (bcc) β-phase. Acicular α-phase forms by nucleation and growth along preferred crystallographic planes in the β-phase during controlled cooling of the part. In some instances, the acicular α-phase grows in a Widmanstätten structure. In contrast, martensitic α-phase (or, α’) is formed by diffusionless transformation of the β-phase during rapid cooling. The martensitic α-phase increases the tensile strength but reduces the ductility of the part [14]. α’ may also have a positive effect on the fatigue strength of the part [21]. A previous study of Ti-6Al-4V processed by DED showed that its microstructure is dominated by needle-like α’-phase in a Widmanstätten structure [22]. LENS has already been used to process Ti-6Al-4V and some other titanium-based alloys [9,23,24,25].

Aircraft stainless steels parts require a combination of high strength and high resistance to corrosion and oxidation along with superb heat and fire resistance. Examples include fuel tanks, exhaust components, engine parts, hydraulic systems, and fasteners. LENS has already been used to process several carbon, alloy, and stainless steels [26,27,28,29,30].

Aluminum-based alloys are common in the aerospace industry thanks to their low density, high specific strength, ductility at low temperatures, high corrosion resistance, availability, good processability, and cost-effectiveness. Applications include plane airframes, wings, air foils, forged engine pistons, fuel cells, fuselages, satellite parts, et cetera. In this work we deposited Al 4047 alloy with high Si content (11–13 wt.%), which gives it superior welding performance. Most Al-based alloys are not suitable for AM due to solidification cracks caused by rapid cooling [13]. A previous study has shown that the microstructure of 4047 produced by AM is complex, with equiaxed and columnar dendrites caused by the rapid cooling of the melt pool [31]. LENS has already been used to process some aluminum-based alloys [32,33,34,35,36].

The ability to obtain printed metallic parts with high quality depends on the AM technology, the printing conditions, the geometry of the printed part, and the physical and chemical properties of the metals and their raw powder feedstock. In technical exhibitions, printer manufacturers often focus more on demonstrators made of stainless steels, possibly because they are cheaper and it is easier to obtain printed items with higher quality using this material. Only recently was the first international standard for design for DED published [37]. This design guide provides information about typical characteristics of DED parts and features, insights into the process-based causes of these characteristics, and an understanding of process capabilities and limitations. 

This article presents a comparative characterization study of items either manufactured from scratch from Ti-6Al-4V alloy and 17-4 PH stainless steel or repaired with aluminum 4047 alloy, which are of interest for aircraft applications. Al 4047 was also used for manufacturing the repaired item from scratch. The main objective was to evaluate the LENS process for both manufacturing and repairing of aircraft parts by designing the demonstrators in-house, letting Optomec print them using the company’s recommended procedures, and conducting quality control in-house. The latter was limited to those tests that are typically required by international standards for quality control of wrought and cast alloys and are commonly available: chemical analysis, metallurgical characterization, density, and hardness testing. X-ray microfocus-computed tomography (μ-CT) and surface roughness analysis were also carried out given their extensive use in the quality control of AM parts, non-destructive character, and the important complementary data that they provide, which could indirectly give indications about the expected mechanical integrity of the printed item. Two secondary objectives were to qualitatively compare the level of porosity determined in metallurgical cross-sections and by μ-CT, and to evaluate the possible effect of the geometry of the printed item on the microstructure.

## 2. Materials and Methods 

Some compromises had to be made in the design STL files of the titanium fitting and the repair of the aluminum flange in order to account for limitations of the LENS MR-7 (Omega) system (Optomec, Inc., Albuquerque, NM, USA) in which the demonstrators were deposited. The company’s recommended procedures were thus followed. Printing conditions are summarized in Table 1. In the following subsections each item is discussed separately.

### 2.1. 17–4 PH Stainless Steel Rod

**Figure 1:**
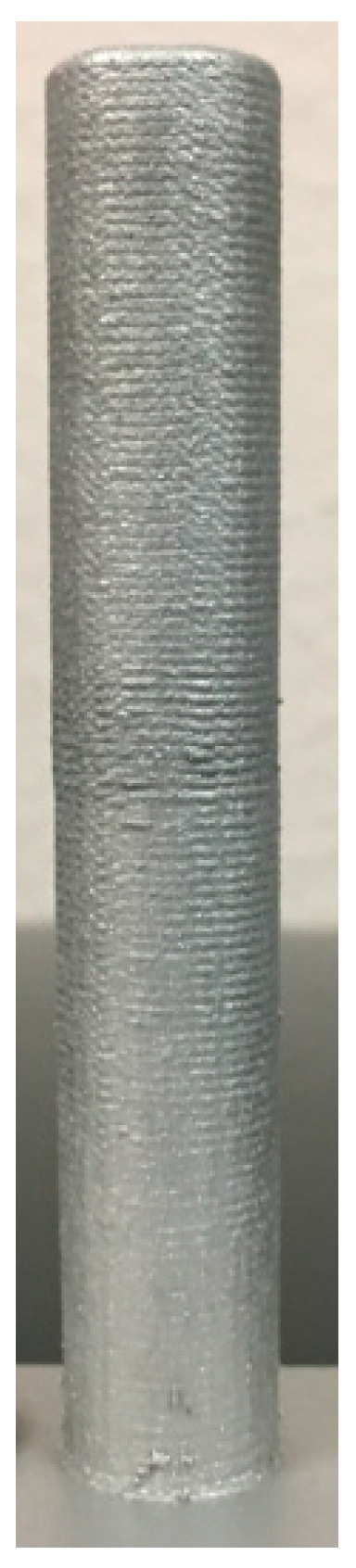
Macroscopic view of a 17-4 PH stainless steel rod with an approximate diameter of 1.27 cm and height of 8.18 cm that was printed by LENS.

The rod was printed using a powder with a particle size range of 44–149 µm (+325–100 Mesh) supplied by Carpenter Powder Products (Bridgeville, PA, USA). The substrate was a general purpose stainless steel, 3.175 mm (0.125 in) thick. The computer-aided design (CAD) file was modified for LENS deposition capabilities and minimal post processing. Deposition was carried out using a standard head. The travel speed was the same for both contour and hatch. The deposition strategy was 0, 90, 180, and 270 degrees sequentially per layer with a hatch spacing of 0.381 mm. Figure 1 shows the printed rod. It has an approximate diameter of 12.7 mm (0.50 in) and height of 81.8 mm, and it took 100 min to print it. Visual inspection indicated good bonding to the substrate, no oxidation, and no evident contamination.

### 2.2. A Fitting Printed of Ti-6Al-4V Alloy

**Figure 2:**
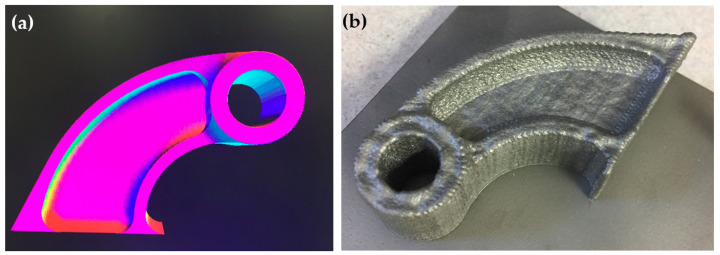
A fitting printed by LENS from Ti-6Al-4V alloy. (**a**) Upright design of the fitting. (**b**) The printed fitting after bead blasting.

The hinge assembly that consists of this fitting and a bushing connects a door to the airframe of aircraft. It was printed according to the model in Figure 2a. A compromise was made in the geometry of the part when designing it in order to account for the limitations of LENS. In addition, it was not possible to build this part straight up since the LENS process is very limited in the fabrication of overhangs [37]. Therefore, it was built with the side down on the substrate, as follows. The manufacturer started with a flat plate that acted as the inner web and thin flange. They then built on both sides of this plate to build up the walls on one side and make the plate thicker on the other side. Figure 2b shows this part built and bead blasted. The final LENS fabricated part had box dimensions of approximately 3.5 × 2 × 1 in. The fitting was printed using a powder with a particle size range of 44–149 µm supplied by TIMET Powder Metals (Morgantown, PA, USA). The substrate was commercially pure (CP) titanium, 6.35 mm (0.25 in) thick. The CAD file was modified for LENS deposition capabilities and minimal post processing. Deposition was carried out using a standard head. The travel speed was the same for both contour and hatch. The deposition strategy was 0, 90, 180, and 270 degrees sequentially per layer with a hatch spacing of 0.508 mm. It took 289 min to print it. Visual inspection indicated good bonding to the substrate, no oxidation, and no evident contamination.

### 2.3. Repair of a Housing Made of Cast AA3xx Aluminum with Aluminum 4047

**Figure 3:**
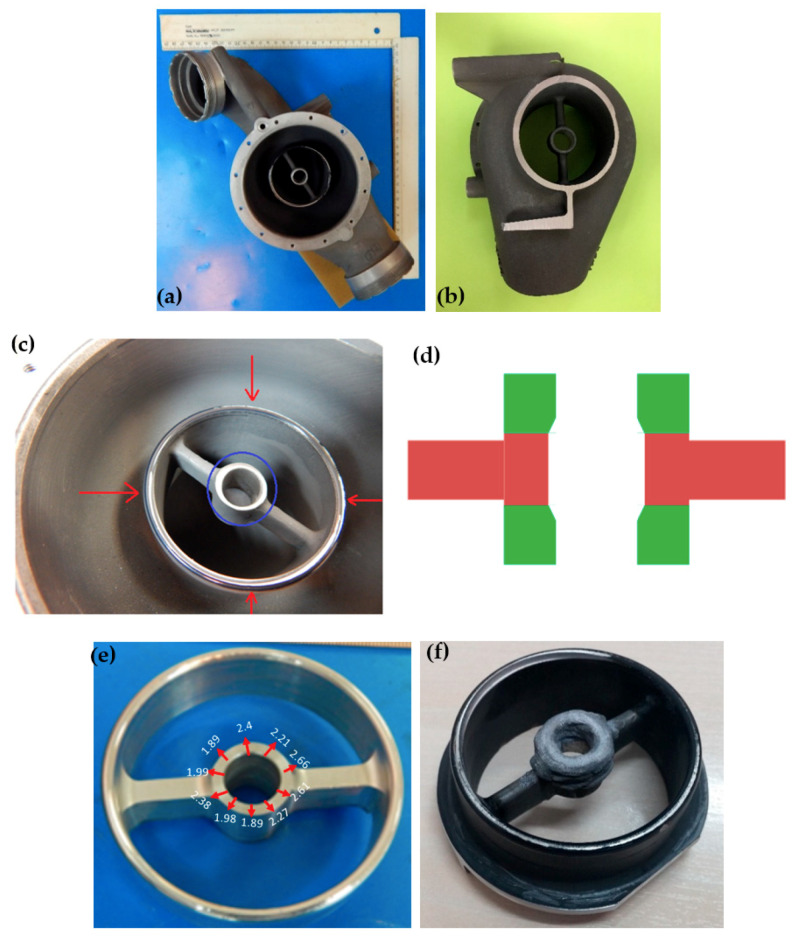
A housing made of cast AA3xx aluminum alloy repaired using LENS and Al 4047 alloy. (**a**) General view, front side. (**b**) Flange back-side opening. This section weighs 1.01 kg. (**c**) Worn area requiring repair. Red arrows mark the border to be repaired according to the model. The blue circle marks the borehole to be rebuilt. (**d**) A side view sketch of the area that was machined prior to repair is colored green. (**e**) Varying wall thickness of the repair housing after grinding, prior to LENS deposition. (**f**) The part after LENS repair.

This pressure operated ram air control valve housing mechanically protects the bore from wear. It is made of cast AA3xx aluminum alloy. A general front side and back side view of this part is given in Figure 3a,b, respectively. Figure 3c shows a zoom-in of the worn area that required repair. It is a challenge to add material with LENS along the entire inside of the bore due to its small diameter. Therefore, Optomec suggested milling both ends of the bore down to the supports, building out both ends up again with a smaller internal shoulder than was necessary, and then boring the shoulders back to the original bore diameter. The shoulder structures on the top and bottom thus provided the bearing surfaces for the piece rotating inside the bore. Figure 3d shows a sketch of the area that was machined prior to rebuilding with the LENS process. After machining, a tool path file was constructed to match the repaired area as closely as possible. This, however, was a challenge since the wall thickness of the circular repair area was varying and the alignment of the laser spot with the repair area was not precise. Figure 3e shows the varying wall thickness as measured using a vernier caliper. With the hand-written tool path file, LENS deposition was done in a controlled argon environment with oxygen content at approximately 6 ppm. The repair material was Al 4047 powder with a particle size range of 44–149 µm supplied by Valimet, Inc. (Stockton, CA, USA). Deposition was carried out using a standard head. The travel speed was the same for both contour and hatch. The deposition strategy was interior contour and exterior contour lines only (namely, hatching not applicable). It took approximately 15 min for the small area-specific repair, and approximately 105 min for the large repair geometry demonstration. Visual inspection indicated good bonding to the substrate, no oxidation, and no evident contamination. Figure 3f shows the repaired area. It is evident from this figure that there was considerable overbuilding and misalignment during the LENS repair process.

**Figure 4:**
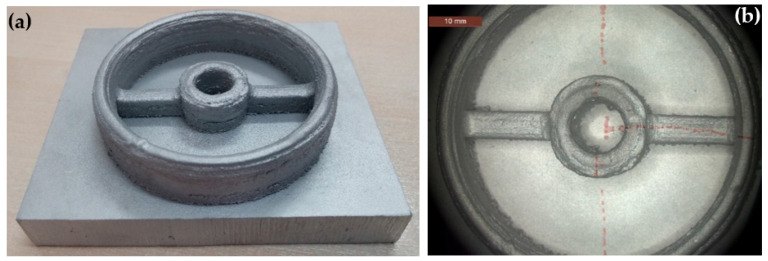
Deposition from scratch of a housing made of Al 4047 alloy by LENS. (**a**) Charge coupled device (CCD) camera view, (**b**) stereomicroscope view.

In order to prove that Al 4047 alloy can be deposited using the LENS process, a tool path file was constructed of only the inner circular area and the outer circular area that were then deposited on a flat aluminum substrate. Figure 4 shows this deposition, which was carried out at the same parameters as listed above. Deposition took approximately 105 min.

### 2.4. Characterization of the LENS Deposited Materials

Chemical analyses of the as-deposited rod and fitting materials were conducted by a metal analysis optical emission spectrometer (Vario Lab, Belec Spektrometrie Opto-Elektronik GmbH, Georgsmarienhütte, Germany). The results were compared to the chemical composition of wrought parts. Local chemical analysis of the repaired area of the aluminum housing was done by energy dispersive X-ray spectroscopy (EDS, model UltraDry, Thermo Scientific, Madison, WI, USA) inside the scanning electron microscope (SEM, JEOL JSM–7000F, Tokyo, Japan). A Leica DFC420 stereoscope was used to examine all items for surface roughness and visible surface defects. Metallurgical cross-sections were prepared at several locations in the stainless steel rod, titanium fitting, and printed aluminum housing from Figure 4. Defects evaluation and micro-hardness testing were conducted on non-etched sections, whereas microstructure analysis and complementary defect analysis were conducted on chemically etched sections. Metallographic cross-sections were prepared by first cutting the metal samples with a Struers Labotom-3 machine, mounting in resin by Buhler Simplimet XPS1 machine, grinding with Buehler AutoMet 300 on 120, 320, and 800 grit SiC papers, and then polishing with Struers LaboPol-5 with 15, 6, and 1 µm diamond pastes on MD Pan polishing pads. Fry’s reagent, Kroll’s reagent, and Keller’s reagent were used as chemical etchants for stainless steel, titanium, and aluminum samples, respectively. The cross-sections were examined under a Reichert-Jung MeF3 light microscope (Leica Microsystems GmbH, Wetzlar, Germany). Micro-hardness tests were conducted using a MicroMet 1600–6400 tester (Buehler, Lake Bluff, IL, USA), Vickers technique, and a load of 200 g. The density of the printed materials was measured using the Archimedes technique. The samples were weighed both in air and in deionized (DI) water at room temperature (without adding a surfactant). An AY220 electronic balance (Shimadzu Corp., Kyoto, Japan) with readability of 0.1 mg was used for this purpose. 

The items to be scanned were placed on a polystyrene jig on top of the μ-CT 5-axis stage. The deposited items were analyzed by μ-CT for defects and internal porosity, either as a whole item or as cut pieces for bulk analysis. A Phoenix v|tome|x m 240 system (Waygate Technologies, Wunstorf, Germany) with detail detectability of less than 1 µm was used for this purpose, and the scanning parameters were voltages of 180–220 kV, current of 50–480 µA, voxel size of 33–87 µm for the large samples and approximately 9–10 µm for the small cut samples, and a beam filter made of either Cu or Sn in varying thicknesses of 0.1–2.0 mm. The dynamic 41|200 large area detector timing was between 333 and 1000 ms. During the scan, the sample was rotated 360°, and approximately 3000 images were acquired, at each position—3 averages and 1 skip in each angular position (i.e., a total of 12,000 frames, of which 9000 were effective). The titanium part was scanned only after removal from the build plate due to technical limitations related to its size and wall thickness. Yet, its dimensions were compared to the dimensions of the designed model, employing an optical scanner (Space Spider, Artec3D, Santa Clara, CA, USA) with a 3D accuracy of 0.05 mm and a 3D resolution of 0.1 mm. Comparison of the density values determined by the Archimedes method was made to the density values calculated from μ-CT data, namely based on defects and internal porosity analysis. A VGDefX ver. 2.2 algorithm was used for this purpose. The unitless pore sphericity factors were also calculated by the algorithm, as the ratio between the surface area of a sphere with the same volume as the given pore to the surface area of the pore:

(1) $$\psi =\frac{\pi^{1/3}{\left(6V_{\mathrm{pore}}\right)}^{2/3}}{A_{\mathrm{pore}}}$$

where *V*_pore_ is the pore volume, and *A*_pore_ is the pore surface area. *ψ*-values vary between 1 and 0, the former corresponding to a perfect sphere, while the latter corresponds to a concave, elongated, or very irregular pore shape. 

The surface roughness was measured using a SmartScope CNC 624 multi-purpose measurement system (Optical Gaging Products, Inc., Rochester, NY, USA) with an XYZ scale resolution of 0.5 µm. The motivation was to broaden the availability of testing equipment. Since the use of this system for roughness measurements is novel, the procedure is described in detail herein. The surface of the printed item was first scanned with the SmartScope system. The topography scan file was converted to the dxf file, which was opened using SolidWorks computer-aided design (CAD) software. Next, five linear segments were extracted from the topographic map, and both the peak-to-valley and average peaks were calculated. Next, the center line was determined, and the differences between the peaks and the center line were calculated. These values were substituted into Equation (2) [38]:

(2) $$R_{a}=\left(\frac{1}{N}\right)\sum_{i=1}^{N}\left|Z_{i}-\bar{Z}\right|$$

where $\bar{Z}=\frac{1}{N}\sum_{i=1}^{N}Z_{i}$, *Z*_i_ is the height at coordinate (*x*,*y*), *i* is the serial number of measurement, and *N* is the number of data points in the profile. Both the average and standard deviation of tens of readings per sample were calculated. The measured data points are tabulated in Tables S1–S3 for 17-4 PH stainless steel, Ti-6Al-4V alloy, and Al 4047 alloy, respectively. In order to validate the novel measurement procedure, a calibration curve was first constructed (Figure S1). To this aim, three standard calibration specimens (*R*_a_ = 3.2, 6.3, and 12.5 µm) from Rubert & Co. Ltd. (Cheshire, UK) with measured values within ±10% of the nominal values on the label were used. The nominal values on the label were drawn versus the actual values measured using the above procedure (Table S4), and linear fitting was constructed, thus obtaining the following conversion equation:

(3) $$R_{a,\mathrm{real}}=0.3491\times R_{a,\mathrm{meas}}+1.9473\;R^{2}=0.9915$$

where *R*_a,meas_ is the value measured in practice, *R*_a,real_ is the true value, and $R^{2}$ is the coefficient of determination. In order to further validate the *R*_a_ values obtained using this novel, non-standard procedure, the surface roughness of each printed item was also analyzed using a ContourGT-K1 optical profilometer (Bruker, Berlin, Germany) with white light interferometry (WLI) hardware, 0.1 nm to 10 mm vertical measurement range, and <1 nm vertical resolution. Each scan had a lateral field of view of ca. 1 × 1 mm.

## 3. Results

### 3.1. 17-4 PH Stainless Steel Rod

The chemical composition of the printed rod is shown in Table 2. Except for some negligible impurities of Al and V that might result from contamination in the LENS chamber, the chemical composition satisfies the requirements of the AMS 5643V standard for wrought 17-4 PH stainless steel (SST).

**Figure 5:**
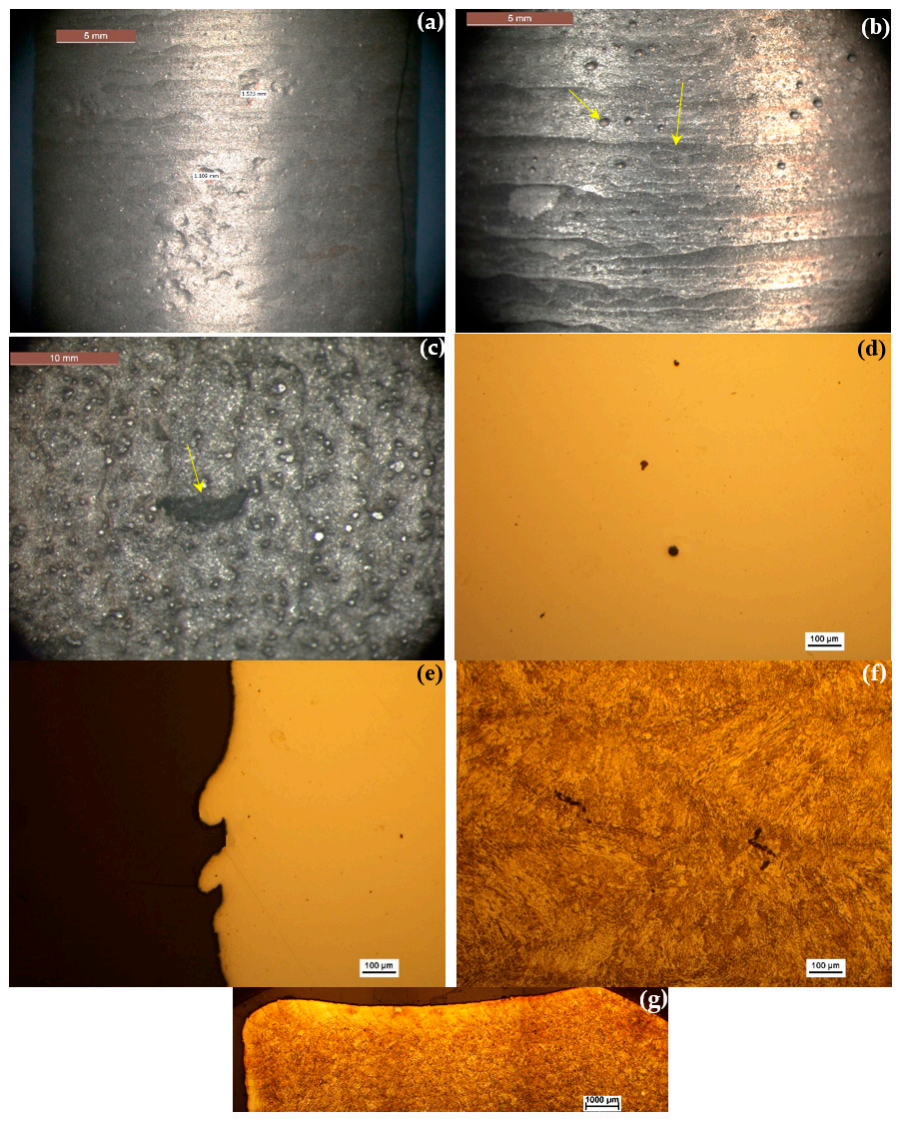
Microscopic characterization of the 17-4 PH stainless steel rod. Stereomicroscope images revealing open porosity (**a**), deposition layers and partially melted powder particles (**b**), and a cavity due to lack of fusion (**c**). Non-etched metallographic longitudinal cross-sections reveal few pores, local chemical inhomogeneity, or both, in the bulk alloy (**d**) and high surface roughness (**e**). A uniform martensitic microstructure (**f**) and overbuild at the top edges (**g**) after chemical etching.

The stereomicroscope images in Figure 5a–c reveal some defects on the surface of the rod. The surface is rough, with some open porosity on the millimeter length scale (Figure 5a), evident deposition layers and some partially melted powder particles (Figure 5b), and a cavity approximately 9.6 mm long (Figure 5c) that reflects a lack of fusion between layers. Figure 5d,e shows metallographic longitudinal (i.e., in the build direction) cross-sections before chemical etching. Some pores, as large as 27.7 μm in diameter (Figure 5d), and some surface asperities (roughness) more than 100 μm deep (Figure 5e) that resulted from incomplete melting of the metal powder, are evident. Such surface roughness is too high for any aerospace application; a post-printing polishing will thus be mandatory. After chemical etching, a uniform, fine martensitic microstructure, and lack of any ferrite or carbides is evident (Figure 5f). The latter conclusion was supported by SEM coupled with EDS mapping not shown herein. This microstructure is typical of steels heat treated at high cooling rates. Pores as long as ca. 90 μm are evident in this cross-section, but they were rare in the bulk sample. Some overbuild is evident at the top edges (Figure 5g). This is typical of samples produced by LENS, which is only a near net shape process, and has been documented for Ti-6Al-4V too [25].

**Figure 6:**
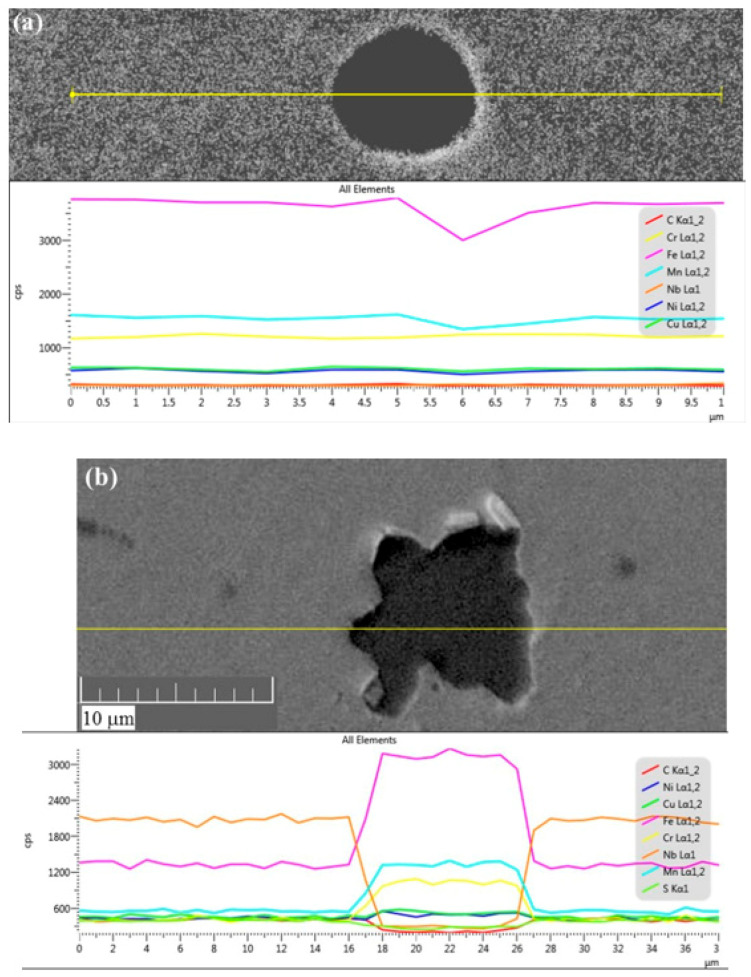
SEM-EDS line scans through typical dark zones in the 17-4 PH stainless steel representing (**a**) pores and (**b**) chemical inhomogeneity.

In order to ascertain that the dark zones in Figure 5d are indeed pores, and not (niobium) carbides with a similar appearance in the optical micrographs that were reported elsewhere for 17-4 PH stainless steel deposited by selective laser melting (SLM) AM [39], a careful SEM-EDS analysis with composition line scans across the dark zones was carried out. Two behaviors were observed: (a) dark zones that either maintain the chemical composition of their surrounding or show some decline in the concentration of certain elements near their walls (Figure 6a), and therefore can be referred to as pores; and (b) dark zones enriched with Fe, Cr, and Mn, and depleted in C and S (Figure 6b). These could neither be referred to as pores nor as (Fe,Cr)_23_C_6_ carbides associated with sensitization [40]. Intermetallic sigma (σ) phases [41,42], massive transformation [43], and δ-ferrite can be ruled out too either from compositional or shape considerations. Hence, we believe that the zones described in (b) result from local chemical inhomogeneity during the deposition process. If so, after solution/homogenization heat treatment they should disappear.

**Figure 7:**
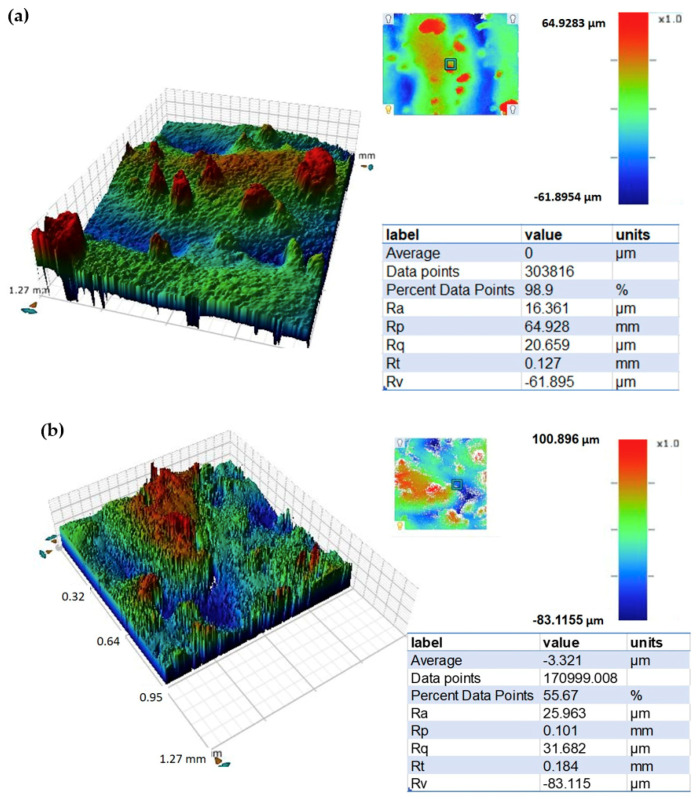

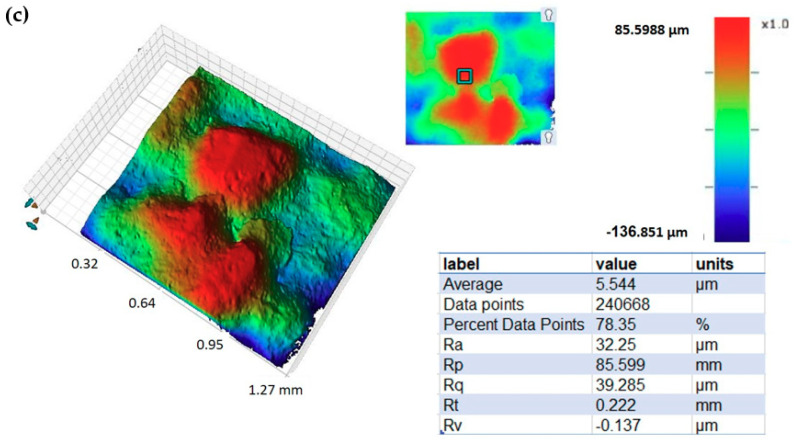
White light interferometry surface scans. (**a**) 17-4 PH stainless steel rod, (**b**) Ti-6Al-4V fitting, and (**c**) Al 4047 housing.

The surface roughness, density, and micro-hardness values of the LENS-processed stainless steel are provided in Table 3. Hardness mean and standard deviation values are statistical analysis of measurements conducted at different locations in the item (including close to the substrate, center, and walls). The Vickers hardness of the stainless steel is equivalent to 40.8 ± 4.4 HRC and matches the AMS [44] and ASTM [45] standards requirement for 0.5-in diameter wrought 17-4 steel in the H900, H925, or H1025 hardening conditions (numbers represent treatment temperature in °F). The measured density of the printed alloy (Table 3) reflects a relative density of ~100% compared to a fully dense material. µ-CT scans of cut pieces yielded a similar density value of 99.98%. The mean *R*_a_ value for the stainless steel rod (Table 3) was between N10 and N11. An excellent match is evident between the values measured with the novel optical gaging procedure and those obtained from WLI. Figure 7a shows a typical WLI scan of the surface of the 17-4 stainless steel.

**Figure 8:**
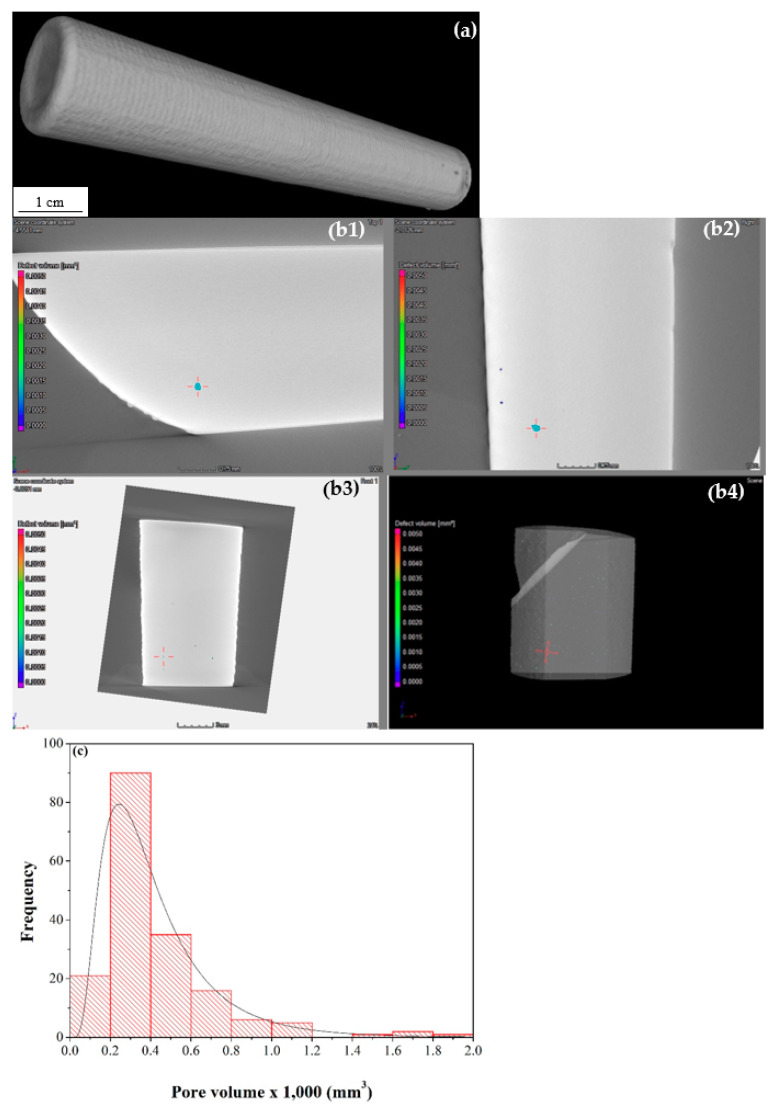
(**a**) μ-CT scan of the steel rod showing no evidence of porosity and other defects. (**b**) μ-CT scan of a cut piece at higher resolution revealing some small defects in the bulk material. (**b1**) XY plane cross-section of the defect. (**b2**) YZ plane cross-section of the defect. (**b3**) XZ plane cross-section of the defect. (**b4**) 3D model with a defect highlighted. (**c**) Pore size distribution in the stainless steel piece with lognormal distribution fit.

The μ-CT scan of the stainless steel rod (Figure 8a) did not reveal defects such as those observed in metallurgical cross-sections (Figure 5) due to the large voxel size (~87 μm). A 3D representation of the scanned model after surface determination was used to differentiate the material from the air. However, a µ-CT scan at a voxel size of 10.01 µm of a small piece extracted from the bulk rod revealed a few defects (Figure 8b), ranging from 51 × 10^−6^ to 18 × 10^−4^ mm^3^ (average: 423 × 10^−6^ mm^3^). Here we present the pore size in volume units rather than in length (radius/diameter) units because we find it most accurate given the non-spherical nature of many pores (the pore sphericity factor values were between 0.45 and 0.68). The darker spots on the right side of Figure 8a were carefully analyzed and were found to be associated with unmelted powder particles on the surface. The pore size distribution in the stainless steel cuts is shown in Figure 8c.

### 3.2. Ti-6Al-4V Fitting

**Figure 9:**
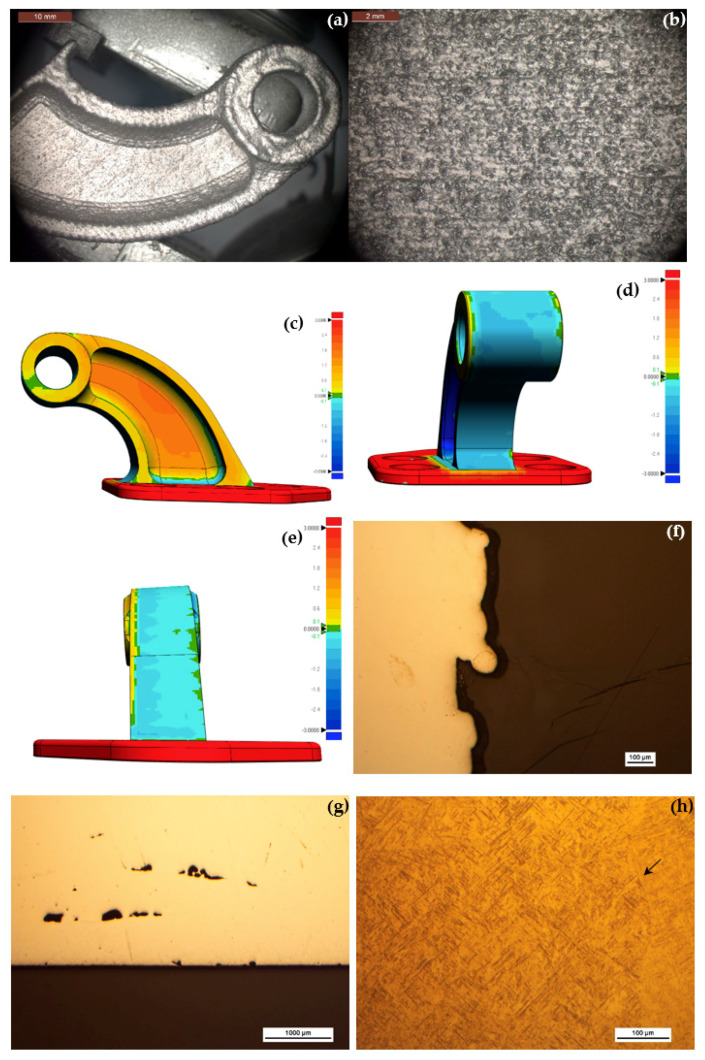

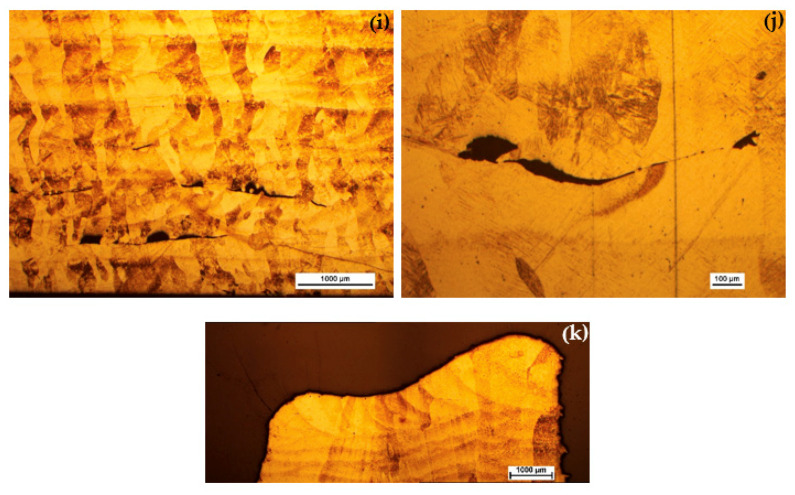
Dimensional and microscopic characterization of the Ti-6Al-4V fitting. Stereomicroscope images revealing distortions and extra build (**a**), surface roughness and partially melted powder particles (**b**). (**c**–**e**) Optical scanner dimension measurements compared to the designed model. Side (**c**), front (**d**), and back (**e**) views. Scale bars in millimeter units; color scheme: red—lower than the model dimensions, green—exactly in the model dimensions, and blue—higher than the model dimensions. (**f**,**g**) Non-etched metallographic cross-sections through the bore area reveal partially melted powder particles on the inner wall of the bore (**f**) and significantly higher porosity in the fitting zone adjacent to the substrate plate (**g**). (**h**) After chemical etching, a fine acicular α’ microstructure and a prior β grain boundary (marked by arrow) are evident. (**i**) A columnar microstructure with the prior grains oriented parallel to the built direction and an interlayer lack of fusion. (**j**) HAZ and interlayer lack of fusion adjacent to the substrate plate. (**k**) HAZ, extra build, and interlayer boundaries around the bore.

The chemical composition of the printed fitting, as shown in Table 2, satisfies the requirements of ASTM F2924 standard for Ti-6Al-4V Grade 5 processed by powder bed fusion (PBF) AM. Figure 9a,b shows stereomicroscope images revealing distortions, extra build at the edges (in particular, around the bore), partially melted powder particles, and surface roughness that seems to be higher than in the case of the 17-4 PH steel (supported by the measured values in Table 3). Figure 9c–e compares the dimensions of the as-printed item to those of the designed model following, based on optical scanner measurements. While the side view shows noticeable underbuild, the front and back views show noticeable overbuild. Figure 9f,g shows two metallographic cross-sections through both sides of the bore before chemical etching. Partially melted powder particles on the inner wall of the bore (Figure 9f) and significantly more pores (as long as ca. 290 μm) in the fitting zone adjacent to the substrate plate (Figure 9g) are evident. We have found the latter to be typical of LENS regardless of the item geometry and material (see also [36]). As in the case of the 17-4 PH steel, a post-printing polishing is mandatory for any aerospace application, to reduce surface roughness. After chemical etching, a fine acicular martensitic α’ arranged in a Widmanstätten microstructure is revealed at all locations in the build, along with prior β grain boundaries (Figure 9h). This microstructure is indicative of the high cooling rates [49,50]. At lower magnification, both a columnar microstructure with the prior grains oriented parallel to the built direction (due to heat extraction from the substrate during solidification) and an interlayer lack of fusion are evident in some zones (Figure 9i). A heat affected zone (HAZ) and interlayer lack of fusion are more noticeable adjacent to the substrate plate (Figure 9j). HAZ, extra build, and interlayer boundaries are noticeable around the bore (Figure 9k). The Vickers hardness of the LENS deposited titanium alloy (ca. 39 HRC, see Table 3) falls between the typical values for annealed or solution and aged wrought Ti-6Al-4V [49]. The measured density of the printed alloy (Table 3) reflects a relative density of 99.89% compared to a fully dense material. µ-CT scans of cut pieces yielded a similar density value of 99.91%. The mean *R*_a_ value for the titanium fitting (Table 3) was between N9 and N12. The values obtained from WLI are closer to the upper limit of the range obtained with the novel optical gaging procedure. Figure 7b shows a typical WLI scan of the surface of the Ti-6Al-4V fitting.

**Figure 10:**
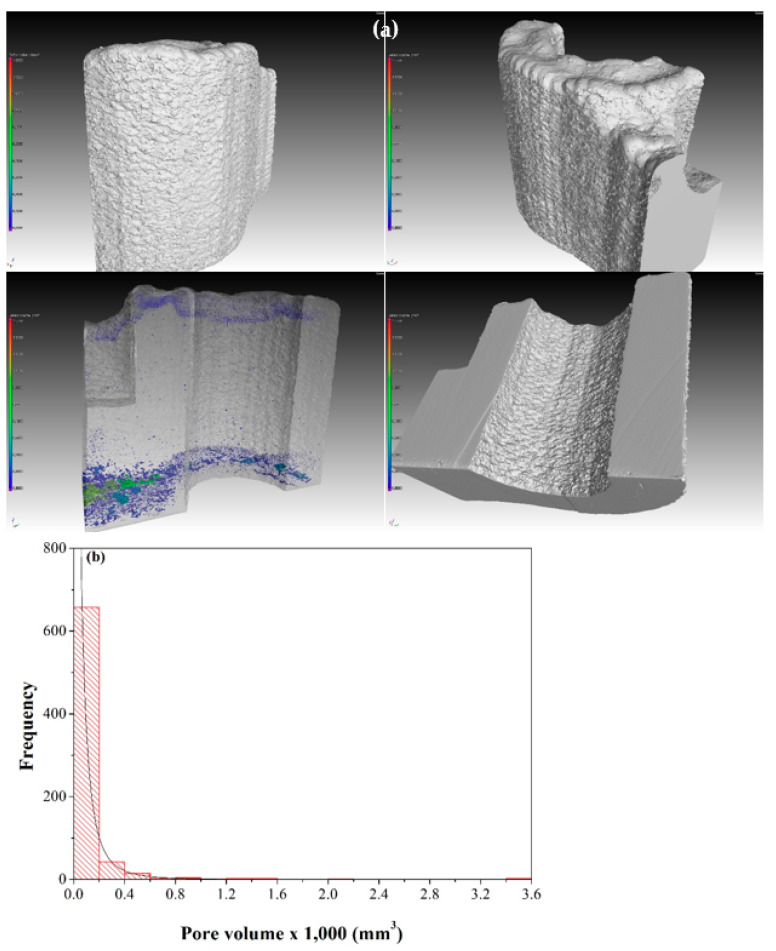
(**a**) A typical μ-CT scan of the cut Ti-6Al-4V fitting at 19.5 μm voxel size from different angles. (**b**) Pore size distribution in pieces of the titanium fitting with lognormal distribution fit.

Due to the large size of the base plate on which the Ti-6Al-4V fitting was deposited, it was necessary to first remove the Ti-6Al-4V fitting from the plate and then cut it into pieces in order to scan at sufficiently high resolution (voxel sizes of 19.5 μm and 9.01 μm for large and small pieces, respectively). A typical μ-CT scan of a cut piece of the Ti-6Al-4V fitting is shown in Figure 10a from different angles. Note the higher density of defects at the interface with the base plate as well as in the top layers of the build. In the large pieces analyzed, the pore volume ranged from 67 × 10^−6^ to 0.5364 mm^3^ (average: 4708 × 10^−6^ mm^3^), and the pore sphericity factor was in the range 0.17–0.74. In the small pieces analyzed, pore volume ranged from 6 × 10^−6^ to 0.0035 mm^3^ (average: 99 × 10^−6^ mm^3^), and the pore sphericity factor was in the range 0.22–0.70. While the latter is not significantly different to the large pieces, the smallest pore detected is approximately one order of magnitude smaller than the large pieces due to the smaller voxel size. The pore size distribution in the titanium cuts is shown in Figure 10b.

### 3.3. Al 4047 Housing Deposited from Scratch

**Figure 11:**
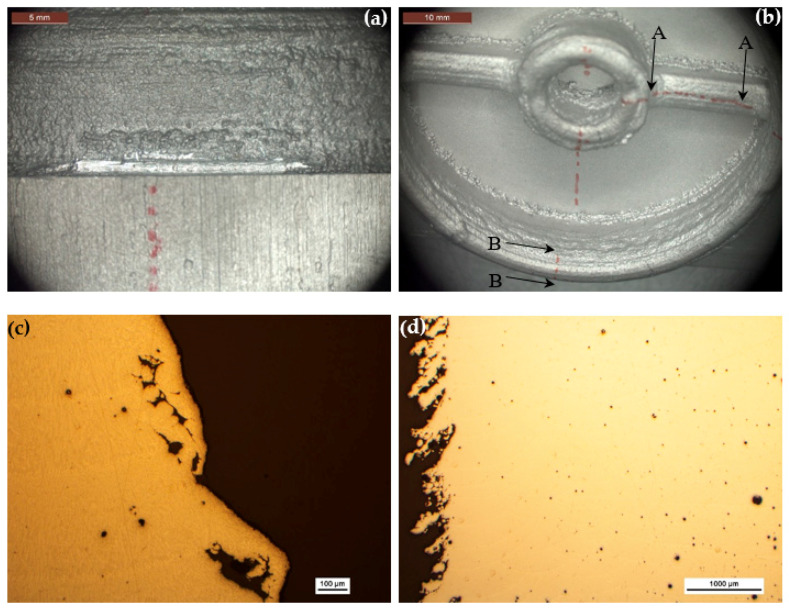

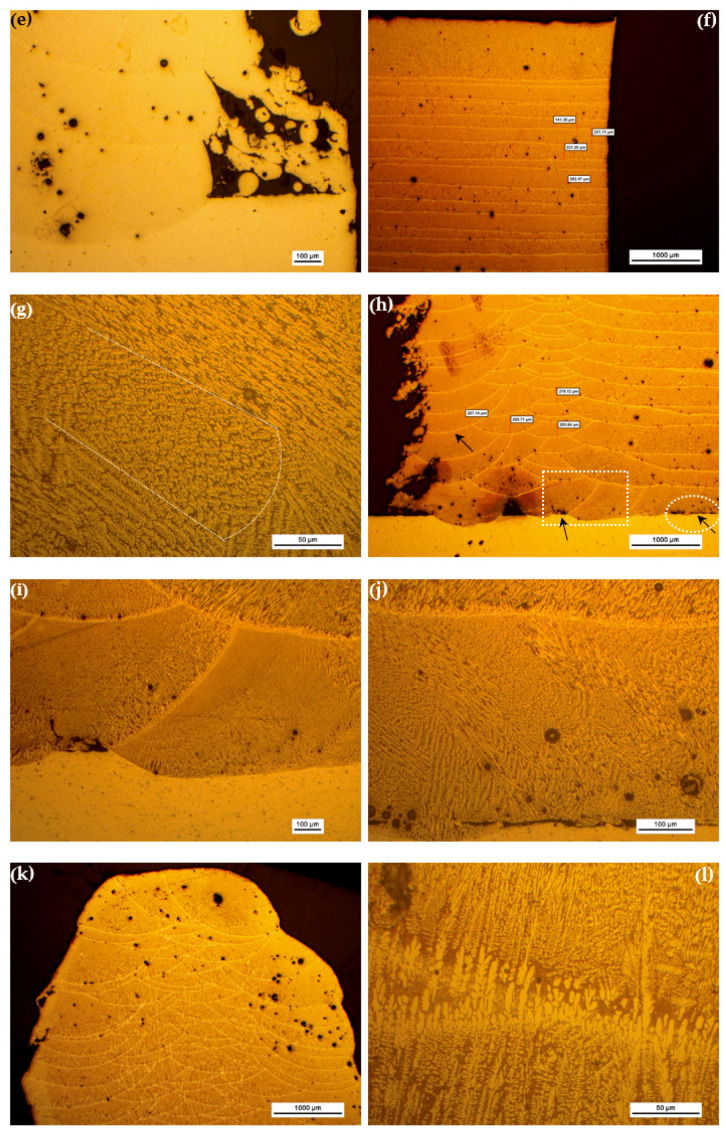
Microscopic characterization of the Al 4047 housing. (**a**,**b**) Stereomicroscope images revealing deposition layers, high surface roughness, and partially melted powder particles both on the outer wall (**a**) and the inner wall (**b**). These particles are accumulated preferentially at the interface between the substrate plate and the housing build. (**c**) Metallurgical cross-section in the A-A orientation marked in (**b**). (**d**) Metallurgical cross-section in the A-A orientation marked in (**b**). (**e**) Metallurgical cross-section in the B-B orientation marked in (**b**). (**f**) Deposition layers and porosity in the A-A cross-section. (**g**) Colonies of dendrites in different orientations. (**h**) The inner wall of the bore and the interface between the substrate plate and the housing build in the A-A orientation. (**i**) The zone marked by a dashed rectangle in (**h**) at higher magnification. (**j**) The zone marked by a dashed ellipse in (**h**) at higher magnification. (**k**) The interpass structure and higher level of porosity near the upper zone of the cross-section in the B-B orientation. (**l**) Dendrites with different sizes.

The chemical composition of the printed housing, as shown in Table 2, satisfies the requirements of AMS 4185F for Al 4047 brazing alloy. From the stereomicroscope images in Figure 11a,b as well as from Figure 4 and Table 3 it is clear that the aluminum item is significantly rougher than both the stainless steel and the titanium parts. The surface density of partially melted powder particles is higher in the aluminum part. These particles tend to accumulate near the interface between the substrate plate and the housing build. Deposition layers are also evident. Figure 11c–e shows metallographic cross-sections before chemical etching, in the A-A (c,d) and B-B (e) orientations marked in Figure 11b. HAZ and large pores along the walls are evident in Figure 11c. High surface roughness, both unmelted and partially melted powder particles on the walls, and high level of porosity in the bulk alloy are apparent in Figure 11d. HAZ, deposition layers, higher level of porosity, and accumulation of both unmelted and partially melted powder particles at the interface between the substrate plate and the outer wall of the housing build are apparent in Figure 11e. After chemical etching, deposition layers and porosity are apparent in the A-A cross-section (Figure 11f). At higher magnification, colonies of dendrites in different orientations are apparent (Figure 11g). The dendritic structure is characteristic of rapid solidification of aluminum. As no hatching was applied in the deposition of this item (see Section 2.3), these orientations should be related to the characteristics of the solidification per se, and not to the laser scan methodology. Figure 11h shows the inner wall of the bore and the interface between the substrate plate and the housing build in the A-A orientation. HAZ, both layer and bead boundaries, lack of fusion, cracking at the interface between the substrate and the housing, and porosity are all apparent. The zone marked by a dashed rectangle is shown in Figure 11i at higher magnification, and similarly the zone marked by a dashed ellipse is shown in Figure 11j. Both spherical pores and cracking between the base plate and the housing build are apparent in Figure 11j. Figure 11k shows the bead-boundary structure and higher level of porosity near the upper zone of the cross-section in the B-B orientation. Figure 11l shows dendrites with different sizes in cross-section. 

Note that in Figure 11h,i,k, the bright circular lines represent the bead boundaries while the bright near-horizontal lines are the layer boundaries. From Figure 11h it is apparent that the layer thickness is not uniform, although laser deposition parameters were kept constant throughout the build. The circular nature of the bead boundary is evident in a section perpendicular to the laser scan direction and is related to the laser track pattern and the near-spherical shape of the melt pool [51].

The surface roughness, density, and micro-hardness values of the LENS-processed Al 4047 are listed in Table 3. The average hardness is comparable to 88.4 ± 3.4 VHN measured in the columnar dendritic structure above the layer/bead boundaries, slightly higher than 86.5 ± 2.3 VHN measured in the equiaxed dendritic structure below the layer/bead boundaries, and higher than 77.5 ± 3.1 VHN measured in the bead boundaries of laser deposited Al 4047 [51]. In another study, of blown powder deposition of thin wall structures of Al 4047 on Al 2024 substrate, a higher hardness of 116 VHN was reported, which was attributed to the lack of porosity [52]. The measured density of the printed alloy (Table 3) reflects a relative density of 99.59% compared to a fully dense material. For comparison, the density of cut pieces based on µ-CT analysis was 98.59%. The mean *R*_a_ value for the aluminum housing (Table 3) was between N10 and N12, and higher than that of the other two materials. A reasonably good match is evident between the values measured with the novel optical gaging procedure and those obtained from WLI. Figure 7c shows a typical WLI scan of the surface of the Al 4047 housing.

**Figure 12:**
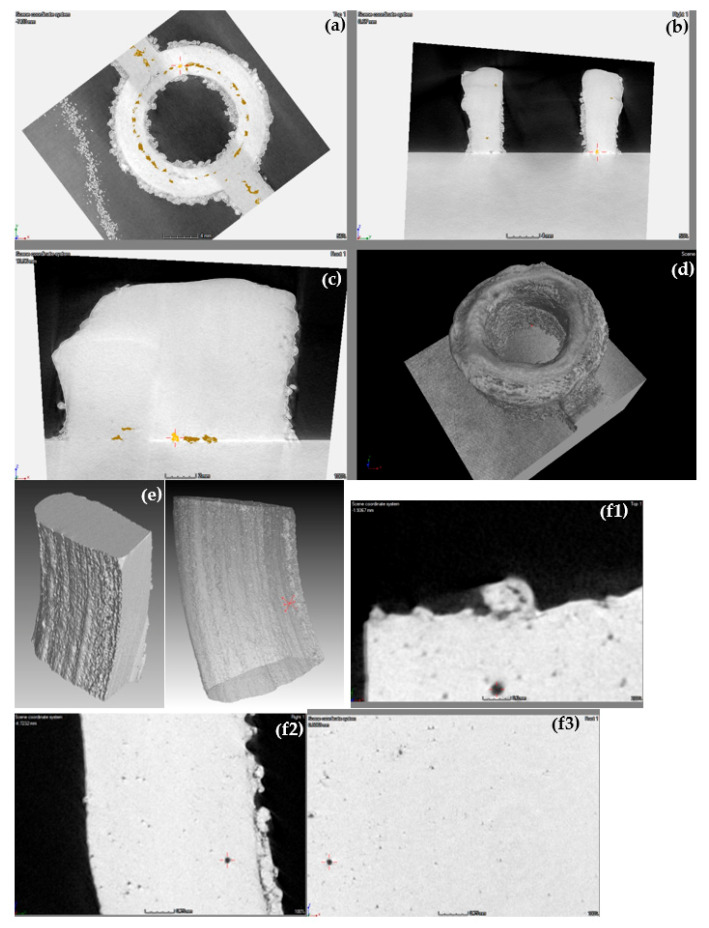

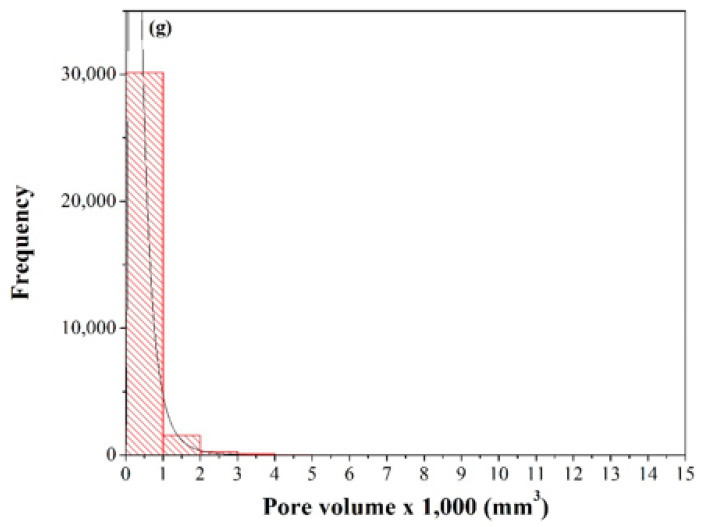
(**a**–**d**) X-ray µ-CT scan of the aluminum housing. Yellow areas mark possible voids. A higher density of defects at the interface between the substrate plate and the housing build is evident from (**c**). (**e**,**f**) µ-CT scanned pieces cut from the housing and scanned at higher resolution. Subfigures f1–f3 show the surface of the model and defects at different cross-sections (XYZ planes). (**g**) Pore size distribution in pieces of the aluminum housing with lognormal distribution fit.

Figure 12 shows selected images from the µ-CT scan of the Al 4047 housing. Defects are apparent, both within the bulk part and at the interface between the part and the substrate plate. The volume fraction of defects is significantly higher in the aluminum housing compared to the stainless steel rod and the Ti-6Al-4V fitting. Metallurgical cross-sections (Figure 11) revealed similar defects in the same regions, thus validating the findings of the µ-CT scan. Figure 12e,f shows µ-CT scans of cut pieces at higher resolution (voxel size 10.2 μm). Partially melted zones are evident. In the whole aluminum housing (voxel size 30.0–33.6 μm), the pore volume ranged between 0.0004 and 0.3239 mm^3^ (average: 0.03859 mm^3^), in other words, much larger than in the other two materials, and the pore sphericity factor was in the range 0.22–0.81. In comparison, in the cut pieces, the pore volume and the pore sphericity factor were in the ranges 22 × 10^−6^–0.0141 mm^3^ (average: 411 × 10^−6^ mm^3^) and 0.18–0.71, respectively. The pore size distribution in the small pieces of the aluminum housing is shown in Figure 12g.

## 4. Discussion

In all three materials, microstructures typical of high cooling rates were observed. These microstructures typically combine high strength with low ductility. Preheating of the substrate plate could aid in reducing the cooling rates, thus tailoring the microstructure. The fact that similar microstructures were observed in different regions in the materials (e.g., near the substrate plate and near the top edge) indicates that the very high cooling rate was insensitive to changes in the geometry and dimensions of the build items (at least, in the length scales employed in this work). Another implication could be the possibility of decreasing the number of metallurgical cross-sections necessary for the approval of a prototype.

The hardness values in all three materials are comparable to those of the counterpart wrought alloys (the latter possibly in a hard condition). This may be the result of the high density of the deposited alloys and their fine microstructure. Variation in hardness was observed in different locations within the same sample. Consequently, the standard deviations in hardness values (11.6%, 2.9%, and 11.1% for the stainless steel, titanium alloy, and aluminum alloy, respectively) are considerably larger than those in wrought alloys in general, and in polished samples in particular [53]. In welded alloys, the standard deviation is often larger than in the counterpart ingots. The highest variation in hardness values for the stainless steel likely results from its higher effective hardness value, fewer number of samples, and heat accumulation during build in the *z*-direction which leads to softening. The latter may be minimized using accessories such as melt pool sensor (MPS). The relatively high standard deviation in hardness values implies that the typical sample size (population) should be increased compared to wrought alloys, and that supporting tensile tests should always be sought (the test samples either being printed from scratch or extracted from printed parts).

Figure 2 and Figure 9c–e allow evaluation of the dimensional and form accuracy of the printed Ti-6Al-4V fitting compared to its design model, keeping in mind that because of the rough surface—the computer analysis to obtain flat surfaces introduces some error. On most surfaces the distortion was associated with dimensions that were smaller than in the model. The only exception where there was overbuild was the top surface, perpendicular to the build direction. This phenomenon results from the characteristics of heat dissipation and boundary constraints, as well as the relatively large layer thickness, and is typical of DED [37]. Overbuild at the top edges (Figure 5g) is also characteristic of the LENS process; it was observed both in the 17-4 PH stainless steel rod and in the Ti-6Al-4V fitting (between 200 and 600 μm). It may be possible to minimize the dimensional uncertainty by adding gradually shortened layers at the top edge to the CAD file (thus forming a pyramid-like shape), which will later be removed by milling. It should be borne in mind that DED processes are usually near-net-shape manufacturing processes, and that post-fabrication steps will often be necessary to achieve desired dimensions, tolerances, and surface finishes [37]. In-process machining could also be applied in the case of hybrid (additive-subtractive) systems. ASTM standard F3413 provides some guidelines to avoid distortion, for example, to use pre-heating of the substrate [37]. Underbuild might result in reduction in mechanical properties such as yield strength or ultimate tensile strength. The anisotropic geometrical distortion should already be accounted for in the design model, whenever possible. However, this might increase both the powder cost and waste generated in the process.

Both partially melted and unmelted powder beads were observed on the surfaces of all items, regardless of the material and geometry (although in aluminum, with its lower melting temperature, they tended to be more partially melted). This is a known phenomenon in AM in general, and in DED in particular; it has undesirable effects on part properties [54]. Partially melted and unmelted powder beads increase the surface roughness. The size of the deposited beads affects the layer thickness, a major variable that influence the minimum surface roughness that can be achieved [37]. Therefore, parts manufactured by LENS for aerospace applications should undergo surface finishing prior to use. Complementary subtractive processes, such as machining, abrasive blasting, vibratory finishing, flow grinding, and electropolishing [38], enable improving of the surface roughness. However, an appropriate machining allowance should be provided for this purpose [37]. The particularly poor surface finish on the inner walls of bores should be noted. Complementary surface finishing will be essential, in particular when a bushing or a dynamic component such as a shaft is to be installed. This should already be taken into account in the design stage, both in regard to tolerances and to access to bores in the surface finishing stage.

μ-CT and the Archimedes methods are commonly used to determine the relative density and porosity of bulk materials, including AM alloys. The Archimedes method does not give any information on pore characteristics, and might be influenced by surface roughness or open pores [36]. On the other hand, μ-CT analysis can be influenced by the X-ray beam scattering and the definition of image processing parameters. On one hand, μ-CT analysis might yield a higher density value than the real one due to resolution limit; on the other hand, the higher occurrence of defects such as pores and lack of fusion at the interface between the substrate plate and the build (see Figure 9g,j, Figure 11f, and Figure 12c) or from the recast layer formed on samples manufactured by electrical discharge machining (EDM) [36] might lower the density value determined by μ-CT analysis. The densities of all three alloys deposited by LENS in this study were high (namely, between 98.59% and ~100% compared to a fully dense material). It has been reported [36,37] that the density of materials printed by DED is often higher than the counterpart materials printed by PBF. Altogether, there was a good match between the density values measured in this study by both μ-CT and the Archimedes method. There was also a good correlation between the type of defects detected by μ-CT and by metallography. For both the Ti-6Al-4V fitting and the Al 4047 housing it was evident that reducing the voxel size in μ-CT by a factor of ca. 2–3 allowed detection of pores with volumes smaller by a factor of ca. 10–20. This should be taken into consideration, for example, when trying to match porosity analysis based on metallurgical cross-sections to that based on μ-CT. The detection capabilities of X-ray CT could be improved further by using nano-focus CT and detectors with smaller pitch size. Furthermore, for the best, most representative μ-CT analysis, detaching the build from the substrate plate before analysis is carried out is recommended.

The pore sphericity factors based on μ-CT analysis were 0.45–0.68 for the 17-4 PH stainless steel rod, 0.17–0.74 for the Ti-6Al-4V fitting, and 0.18–0.81 for the Al 4047 housing. The narrower range of values in the case of the stainless steel may be attributed both to the material and to the less complex geometry of the printed item. There is no clear definition of the correlation between sphericity value and defect type. Yet, Snell et al. [55] recently showed, based on clustered results, that sphericity factors lower than 0.6, higher than 0.7, and higher than 0.92 represent an irregular shape that may be associated with lack of fusion, keyholes (usually, the result of high energy causing localized vaporization), and gas porosity, respectively. Irregular pores may also be attributed to partially melted powder particles resulting from insufficient laser energy [56]. Another cause of irregular defect formation in low-energy processing of Al alloys is failure to effectively eliminate oxide films that cover the powder and prevent consolidation [56]. The sphericity values measured in this work support defects such as lack of fusion and partially melted powder particles that were observed both in metallurgical cross-sections and in μ-CT images. In addition, for all three materials, they indicate that gas porosity is not involved. Finally, it should be noted that crack-like voids, such as those with sphericity lower than 0.7 and high aspect ratios, might degrade the mechanical properties if they are oriented perpendicularly to the loading direction [57].

Porosity, chemical inhomogeneity, surface roughness, and residual thermal stresses could all increase the susceptibility of AM metals and alloys to corrosion [58,59,60]. Inherent porosity could also increase the susceptibility of iron-based alloys, including steels, and other materials with positive heat of hydrogen solution to hydrogen embrittlement in a high pressure bubble formation mechanism [61]. The thermal condition, and the related size, density, and degree of coherency of precipitates, of precipitation hardening steels could affect both hydrogen diffusivity and hydrogen solubility in the steel [62], for example, due to elastic stress fields around precipitates [63]. The specific microstructure and interphase boundaries could also affect the susceptibility to hydrogen-induced cracking. All of these should be borne in mind when comparing the microstructures of AM alloys to those of the counterpart wrought (and cast) alloys, as they might affect the environmental degradation of the material in service and maintenance.

Both the static and the dynamic properties of DED materials are typically superior to those of the counterpart PBF materials, and can be the same as or even better than those of the wrought material counterparts after post-processing [37]. Usually, hot isostatic pressing (HIP) or annealing post-processes are not required to meet the ASTM standard requirements for elongation [37]. However, these properties depend to some extent on the material selected, process settings, and part shape [37]. The use of digital image correlation (DIC) with tension tests [36,64,65] is recommended for measurement of the static mechanical properties, while dynamic pulse-echo ultrasonic non-destructive test (NDT) with a time-of-flight (TOF) sound velocity analysis is recommended for measurement of the elastic constants [36].

There are only few reports available on the processing of 17-4 PH (AISI 630, UNS S17400) by LENS [18,66], but some useful information can be gained from reports of its processing by SLM [39,67,68]. It was reported, for example, that powder porosity had a more significant contribution to intralayer porosity than deposition conditions [18]. Ultrasonic vibration during deposition was found to enhance the process, geometrical and microstructural characteristics of this steel, namely, increase the powder utilization efficiency, decrease the surface roughness, increase the molten pool dimensions, reduce porosity and micro-cracking, and refine the grain size, thus leading to superior hardness and tensile properties [66].

In the case of Ti-6Al-4V, distortions, extra build at edges, partially melted powder particles, HAZ, and interlayer lack of fusion were observed. Defect concentration was significantly higher adjacent to the substrate plate. Both the surface roughness and the level of porosity were higher than in the case of the 17-4 PH steel. These differences are most likely related to the material chemistry and physical properties, although the effect of geometry and its complexity cannot be excluded. The fine acicular martensitic α’ arranged in a Widmanstätten microstructure observed in this study should provide high tensile strength [14] and fatigue strength [21], but also reduced ductility [14]. In practice, a post-processing thermal treatment may be necessary to improve the ductility. Ti-6Al-4V is the most commonly processed titanium alloy, both by DED and by PBF. Its processing by LENS has also been reported [9,23,24,25,69,70,71,72,73,74,75,76,77,78,79,80,81,82].

There have been some contradictions regarding the static and dynamic mechanical properties of LENS-processed Ti-6Al-4V compared to its wrought and PBF counterparts. For example, a shorter fatigue life of the LENS alloy than of the wrought alloy has been related to microstructure and porosity [69]. A complexity arises, however, from the effect of pore geometry and location on the extent to which the fatigue life is reduced. Consequently, the scatter in fatigue data might be high and the uncertainty be increased. Optimization of the LENS process to remove defects was thus suggested in order to improve the mechanical properties. In addition, finer microstructures, consisting of more grain boundaries, could lower the fatigue life at higher strain amplitudes and increase the fatigue life at lower strain amplitudes [69]. In another work [76,78], LENS-fabricated Ti-6Al-4V exhibited significantly lower ductility, similar tensile strength, similar fracture toughness, and similar fatigue crack growth (FCG) threshold values (when processed at high power) compared to an electron beam melting (EBM) processed alloy. Ti-6Al-4V LENS-processed at high power had higher FCG threshold values than Ti-6Al-4V LENS-processed at low power. The LENS-processed alloy showed better low-cycle fatigue (LCF) performance, poorer high-cycle fatigue (HCF) performance, lower threshold, and higher fracture toughness compared to mill-annealed Ti-6Al-4V. These differences were attributed to the lamellar microstructure in the LENS alloy. The α-phase morphology was claimed to be the controlling factor of the FCG behavior of the LENS alloy; deposition at high power yielded coarser α morphology, and consequently, slightly higher FCG thresholds and lower region II FCG rates [78]. In yet another study [79], shorter fatigue lives of LENS-processed Ti-6Al-4V compared to the wrought alloy were attributed to porosity.

On the other hand, Kobryn and Semiatin [80] placed the measured fatigue strengths of LENS-processed Ti-6Al-4V in both the stress-relieved and HIP conditions on the S–N curves of wrought, cast, and cast plus HIP alloys. It was concluded that the static tensile strength and ductility, fatigue strength, and fracture toughness of HIP LENS-processed Ti-6Al-4V compare favorably to those of the wrought alloy. Lack-of-fusion porosity had a significant effect on both the levels and anisotropy of mechanical properties [80]. The yield strength showed a noticeable anisotropy due to residual deposition porosity in the stress-relieved condition, and mechanical and crystallographic texture in both the stress-relieved and HIP conditions [80]. HIP appeared to be effective in healing lack-of-fusion porosity. Prabhu et al. [81] found that in spite of the expectation that partially melted particles at the surface would trigger crack initiation and decrease the fatigue life significantly, LENS-processed Ti-6Al-4V had fatigue life comparable to the wrought alloy, and may be used both for printing from scratch and repair without the need for HIP. Finally, Razavi and Berto [82] found that LENS-processed Ti-6Al-4V had higher fatigue life and fatigue strength compared to its counterpart wrought alloy. This was attributed to the finer grain size in the LENS alloy, which leads to higher fatigue crack nucleation time, and to the basket-weave and columnar prior β grains in its microstructure, which leads to higher degree of tortuosity and, accordingly, more roughness-induced closure effects during crack propagation [82].

The attempt to repair a housing made of cast AA3xx aluminum with Al 4047 failed: there were considerable overbuilding and misalignment during the LENS repair process. Then, the deposition from scratch of the inner and outer circular areas from Al 4047 revealed some of the challenges in AM of aluminum alloys. The layer thickness was not uniform, although laser deposition parameters were kept constant throughout the build. For future repair with Al 4047, we advise prior cleaning of the deposited surface via chemical cleaning (e.g., in phosphoric acid for at least 20 min). In order to minimize the temperature gradient and achieve better connection between the deposited material and the existing part, it could be beneficial to preheat the surface to 150 °C with the laser of the LENS machine. In addition, we advise that the first deposited layers be printed at significantly lower travel speed and energy than the build itself. To avoid lack of fusion or cracking in the outer deposited layers, which determine the surface roughness, a higher laser power should be implemented. In addition, as mentioned before, the alignment of the laser spot with the repair area was not precise. To avoid this problem, a prior CNC machining is recommended to achieve uniform bore radii, along with the use of a precise tool path file instead of a hand-written one.

The surface roughness, surface density of partially melted powder particles, and the content of bulk defects were significantly higher in Al 4047 than those in 17-4 PH stainless steel and Ti-6Al-4V alloy. Partially melted powder particles may result from the high surface reflectivity of the Al powder, for example [36]. Other challenges of laser-beam processing of aluminum alloys are summarized elsewhere [36]. The cracks at the interface between the build and the substrate plate are similar to those often observed in aluminum welding; they were caused by high thermal stresses and mismatches between the first deposited layer and the plate [83]. All of these challenges may explain why LENS-deposited Al 4047 has not been reported before (although Al 4047 has been deposited with several other blown-powder systems [31,51,52,84]).

## 5. Conclusions

Comparative characterization of 17-4 PH stainless steel rod, Ti-6Al-4V fitting, and Al 4047 housing manufactured by laser engineered net shaping (LENS) was conducted. The following conclusions were drawn:

1. Anisotropic geometrical distortion and overbuild at top edges were observed. These should already be accounted for in the design model, whenever possible.
2. In all three materials, microstructures typical of rapid solidification were observed, along with high density, chemical composition, and hardness comparable to those of the counterpart wrought alloys (even in hard condition).
3. The detected defects included partially melted and unmelted powder particles, porosity, and interlayer lack of fusion, in particularly at the interface between the substrate plate and the build.
4. The sphericity values obtained from μ-CT analysis supported the metallographic and μ-CT images well.
5. The standard deviations in hardness values were considerably larger than those in wrought alloys in general, and in polished samples in particular.
6. There was a fairly good match between the density values measured by μ-CT and those measured by the Archimedes method; there was also good correlation between the types of defects detected by both techniques.
7. For both the Ti-6Al-4V fitting and the Al 4047 housing, reducing the voxel size in μ-CT by a factor of ca. 2–3 allowed detection of pores with volumes smaller by a factor of ca. 10–20. This should be taken into consideration when trying to match porosity analysis based on metallurgical cross-sections to those based on μ-CT. For the best, most representative μ-CT analysis, detaching the build from the substrate plate before analysis is carried out is recommended.
8. A novel, non-standard optical gaging procedure for measurement of surface roughness was proposed, using a commercial video and multisensory measurement system with a large measurement range. A fairly good match with values obtained from white light interferometry is shown.
9. The surface roughness of the inner walls of bores was particularly poor.
10. Surface roughness, density of partially melted powder particles, and the content of bulk defects increased slightly from 17-4 PH stainless steel to Ti-6Al-4V, but then significantly increased in Al 4047.
11. The attempt to repair a housing made of cast AA3xx aluminum with Al 4047 failed due to considerable overbuilding and misalignment.
12. Parts manufactured by LENS for aerospace applications must undergo surface finishing prior to use. Appropriate tolerances and access to surfaces for complementary surface finishing should already be taken into account in the design stages.

## Figures and Tables

**Table 1:** Summary of LENS deposition parameters.

Item	Deposited Powder	Laser Power (W)	Powder Feed Rate (g/min)	Travel Speed (cm/min)	Layer Thickness (µm)
Rod	17-4 PH steel	380	26.16	101.6	381
Fitting	Ti-6Al-4V	450	3.78	63.5	381
Housing repair	Al 4047	1000	9.84	76.2	254

**Table 2:** Chemical analysis of the printed items (wt.%) in comparison to standard requirements for the equivalent wrought alloys.

Item	PH Stainless Steel Rod	SST Standard [44]	Ti-6Al-4V Fitting	Ti-6Al-4V Standard [46]	Al 4047 Housing	Al 4047 Standard [47]
Fe	73.643	Bal.	0.395	max 0.30 (±0.10)	0.172	max 0.80
Cr	15.760	15.00–17.5	0.045	–	0.002	–
Ni	4.302	3.00–5.00	0.024	–	0.007	–
Cu	3.992	3.00–5.00	–	–	0.003	max 0.30
Si	0.949	max 1.00	0.019	–	11.23	11.0–13.0
Mn	0.797	max 1.00	–	–	0.001	max 0.15
Mo	0.098	max 0.50	0.07	–	–	–
C	0.034	max 0.070	0.009	max 0.08	–	–
O	–	–	N/A	max 0.20	N/A	–
N	–	–	N/A	max 0.05	N/A	–
H	–	–	N/A	max 0.015	N/A	–
Nb	0.233	5×C–0.45	0.05	–	–	–
P	0.015	max 0.040	–	–	0.005	–
S	0.014	max 0.030	–	–	–	–
Al	0.018	0 (+0.01) [48]	6.28	5.50–6.75	88.43	Bal.
V	0.037	0 (+0.03) [48]	3.74	3.50–4.50	0.011	–
Y	–	–	–	max 0.005	–	–
Ti	–	–	89.37	Bal.	0.009	–
Mg	–	–	–	–	0.005	max 0.10
Zn	–	–	–	–	0.022	max 0.20
Be	–	–	–	–	–	max 0.0008
Other elements	0.108	–	0.208	max 0.40 total, max 0.10 each	0.127	max 0.15 total, max 0.05 each

**Table 3:** Micro-hardness, density, and surface roughness of the as-processed LENS materials.

Material	Roughness *R*_a,real_ (μm) ^§^	Roughness *R*_a_ (μm) ^‡^	Density (g/cm^3^)	VHN *
17-4 PH steel	15.2 ± 8.7	16.00 ± 1.26	7.816 ± 0.240	400.1 ± 46.6
Ti-6Al-4V	18.7 ± 12.6	26.21 ± 4.00	4.425 ± 0.020	382.3 ± 11.1
Al 4047	22.9 ± 12.1	27.71 ± 8.95	2.649 ± 0.110	89.5 ± 9.9

* *n* = 5, 12, and 8 for the steel, titanium, and aluminum items, respectively. **^§^** Optical gaging. *n* = 81, 89, and 56 for the steel, titanium, and aluminum items, respectively. **^‡^** White light interferometry. *n* = 7, 7, and 9 for the steel, titanium, and aluminum items, respectively.

## Supplementary Materials

The following are available online at https://www.mdpi.com/1996-1944/13/18/4171/s1, Figure S1: Calibration curve constructed by drawing the nominal values on the labels of the standard calibration specimens (Ra,real) versus the measured values (Ra,meas), Table S1: Measured surface roughness values (μm) for the 17–4 PH stainless steel rod, Table S2: Measured surface roughness values (μm) for the Ti–6Al–4V fitting, Table S3: Measured surface roughness values (μm) for the Al 4047 housing, Table S4: Measured roughness values (μm) for three standard calibration specimens with different label nominal values.
