# Supplementary material for: Comparative Quality Control of Titanium Alloy Ti–6Al–4V, 17–4 PH Stainless Steel, and Aluminum Alloy 4047 Either Manufactured or Repaired by Laser Engineered Net Shaping (LENS)
Source: Materials (Basel). 2020 Sep 19;13(18):4171. doi: 10.3390/ma13184171 (PMC7560294; doi:10.3390/ma13184171)
Supplement: Supplementary file 1 [file materials-13-04171-s001.pdf]

materials

Supplementary file

Comparative Quality Control of Titanium Alloy Ti–6Al–4V, 17–4 PH Stainless Steel, and Aluminum Alloy 4047 either Manufactured or Repaired by Laser Engineered Net Shaping (LENS)

Noam Eliaz [1,*], Nitzan Foucks [1,2], Dolev Geva [3], Shai Oren [2], Noy Shriki [2], Danielle Vaknin [2], Dimitry Fishman [2], and Ofer Levi [2]

[1] Biomaterials and Corrosion Lab, Department of Materials Science and Engineering, Tel-Aviv University, Ramat Aviv, Tel Aviv 6997801, Israel; nitzanfoucks2@gmail.com (N.F.); neliaz@tau.ac.il (N.E.)
[2] Materials Science and Engineering Division, Depot 22, Israel Air Force, P.O. Box 02538, Tel Aviv, Israel; orenshai20@gmail.com (O.S.); noya.shr@gmail.com (N.S.); daniellva199658@gmail.com (D.V.); dimitry.fishman@gmail.com (D.F.); ofemi6674@gmail.com (O.L.)
[3] Israel Ministry of Defense, Hakirya, Tel Aviv 61909, Israel; dolev24@yahoo.com (D.G.)
* Correspondence: neliaz@tau.ac.il; Tel.: +972-3-640-7384 (N.E.)

2. Materials and Methods

2.4. Characterization of the LENS® Deposited Materials

The surface roughness was measured using a SmartScope® CNC™ 624 multi-purpose measurement system (Optical Gaging Products, Inc., Rochester, NY, USA) with an XYZ scale resolution of 0.5 μm. The measured surface roughness values ($R_{a,meas}$) are tabulated in Tables S1, S2, and S3 for 17–4 PH stainless steel, Ti–6Al–4V alloy, and Al 4047 alloy, respectively. The conversion equation (Eq. (3) in main manuscript) is derived from the linear fitting shown in Figure **S1**.

Table S1. Measured surface roughness values (µm) for the 17–4 PH stainless steel rod.

Line 1	Line 2	Line 3	Line 4	Line 5
36	53	6	23	33
14	66	33	16	38
76	46	31	42	33
35	73	9	44	24
65	38	10	52	43
24	59	27	26	27
61	55	46	52	79
29	60	9	34	44
44	29	24	74	9
51	43	41	21	71
53	7	11	69	24
38	15	29	72	47
40	9	36	23	45
51		16		51
35		37		29
31		30		44
		20		59
		44		40
		21		92
		17		

Table S2. Measured surface roughness values (μm) for the Ti–6Al–4V fitting.

Line 1	Line 2	Line 3	Line 4	Line 5
130	74	72	43	38
50	71	86	54	97
23	20	38	30	98
77	58	20	40	36
44	42	7	65	36
84	11	51	88	86
101	5	75	70	62
40	16	24	54	73
88	38	29	31	95
31	11	12	21	40
39	5	121	44	39
32	68	34	19	57
50	35	12	29	61
28	84	118	32	
25	9	12	36	
23	16	28	24	
55	19	24	60	
19	38	67		
83	50	144		
	27	25		

Table S3. Measured surface roughness values (µm) for the Al 4047 housing.

Line 1	Line 2	Line 3	Line 4	Line 5
23	91	34	80	101
61	57	38	90	57
39	77	65	60	37
46	124	50	125	84
92	39	80	45	86
36	43	53	29	56
44	87	74	59	101
56	52	28	29	43
89	15	53	106	13
	29	109	102	9
			58	36
			38	22
				72
				41
				98

Table S2. Measured roughness values (μm) for three standard calibration specimens with different label nominal values.

**3.2**	**6.3**	**12.5**
2.8	10.6	27.3
4.2	9.9	29.4
5.8	12	32.0
3.2	9.2	35.4
6.2	14.6	32.8
4.8	5.2	34.7
4.2	15.3	25.8
3.8	10.4	35.7
3.8	13.4	25.6
6.2	5.7	29.5
3.8	16.1	26.0
3.2	16.8	32.5
7.8	14.4	33.0
2.8	8.4	34.3
5.6	13.1	33.6
3.8	13.5	33.9
6.7	9.3	30.4
4.4	6.7	30.0
3.4	10.3	34.8
4.9	11.8	33.8
5.3	8.5	30.2
4.3	10.4	28.6
5.4	6.9	30.4
4.3	13.7	30.2
5.8	13.2	33.5
3.3	8.5	30.3
4.9	9.8	32.0
4.4	13.1	32.3
	10.6	
	11.0	
	11.1	

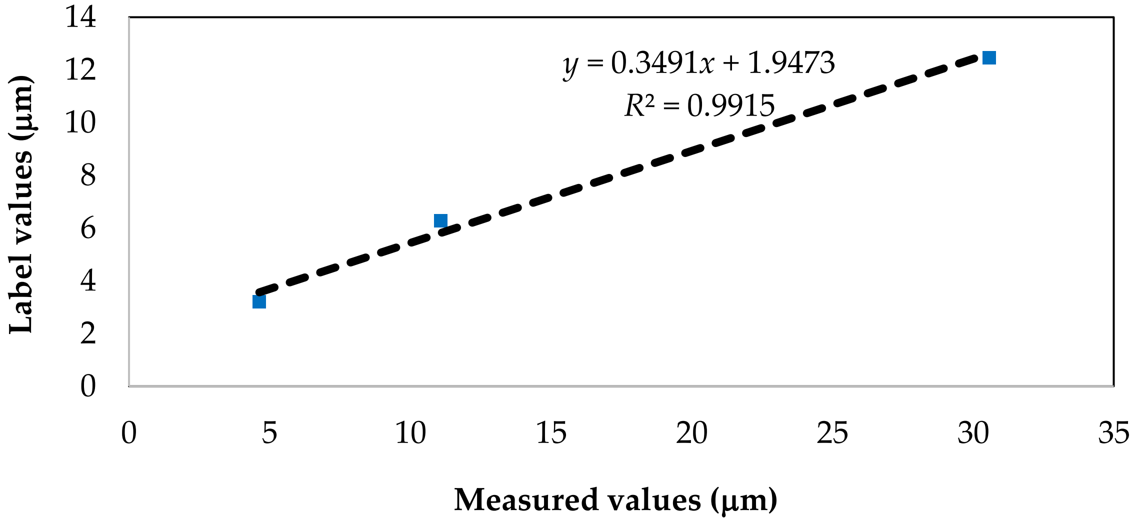

Figure S1. Calibration curve constructed by drawing the nominal values on the labels of the standard calibration specimens ($R_{a,real}$) versus the measured values ($R_{a,meas}$).
